# No added risk: national outcomes of magnetic sphincter explant with hiatal hernia repair and fundoplication

**DOI:** 10.1007/s00464-026-13081-0

**Published:** 2026-07-10

**Authors:** Sienna Wong, Paul Wisniowski, Desmond Huynh, Yufei Chen, Kulmeet Sandhu, Scott Cunneen, Miguel Burch

**Affiliations:** Department of Minimally Invasive Surgery, Cedars Sinai Medical Center, 8700 Beverly Blvd, Suite 795W, Los Angeles, CA 90048 USA

**Keywords:** GERD, Magnetic sphincter augmentation, Hiatal hernia repair, Fundoplication

## Abstract

**Background:**

Magnetic sphincter augmentation (MSA) is a ring of titanium beads that is implanted around the gastroesophageal junction for the management of gastroesophageal reflux disease, providing an alternative to traditional fundoplication. Despite positive outcomes, a subset of patients require device explantation. The safety of patients undergoing explant with revisional surgery remains limited to single-institution experiences. The objective of this study was to compare perioperative outcomes between patients undergoing MSA device explant with revisional surgery and those undergoing primary surgery using a national database.

**Methods:**

The 2017–2023 American College of Surgeons National Surgical Quality Improvement Program registry was queried for patients undergoing MSA removal with concurrent hiatal hernia repair and fundoplication. These patients were compared to a cohort undergoing primary hiatal hernia repair with fundoplication. Propensity score matching was performed in a 1:1 fashion based on baseline demographics, comorbidities, and procedure type to minimize selection bias. Outcomes of interest included operative time, length of stay, thirty-day postoperative complications, readmissions, and mortality.

**Results:**

A total of 49,535 patients underwent foregut surgery during the study period, of whom 40 (0.01%) underwent MSA device explant with revisional surgery. Following propensity score matching, each cohort included 40 patients. Overall demographics between the MSA and primary hernia repair groups were similar with the majority being female (87.5%), mean ages of 58.3 and 58.8 years, and BMI of 29.1 and 28.9 kg/m^2^, respectively. For both groups, the complication rate was 2.5% for readmission requiring revisional surgery, and median length of stay was one day. Median operative time was comparable. No mortalities were reported.

**Conclusions:**

This study represents the largest national analysis of patients undergoing MSA device explant with concurrent revisional foregut surgery. Findings suggest that MSA device removal with hiatal hernia repair and fundoplication is safe, with perioperative outcomes equivalent to those of primary foregut surgery.

Gastroesophageal reflux disease (GERD) is one of the most common gastrointestinal disorders worldwide, affecting over 1 billion individuals with significant quality of life (QOL) and healthcare cost implications [1]. Management of GERD is primarily medical; however, when medical management fails, surgical intervention is indicated. While fundoplication has traditionally been the mainstay for anti-reflux surgery, the introduction of magnetic sphincter augmentation (MSA) has provided an alternative surgical approach. First introduced to the market in 2012, more than 40,000 devices had been implanted with positive long-term outcomes yielding multi-society consensus as a viable alternative for the treatment of GERD [2–4]. The most common side effect of MSA device implantation is dysphagia, estimated to occur in approximately 68% of cases immediately postoperatively, but improves to 11% at 1 year and continues to improve over time [5–7].

A subset of these patients will experience adverse events such as persistent GERD symptoms, intolerable dysphagia, or serious complications such as device erosion or migration that necessitates removal, with rates in literature ranging from 2.2 to 28% [3, 8–10]. When indicated, removal may be combined with hiatal hernia repair (HHR) and fundoplication to maintain anti-reflux control [10, 11].

Revisional foregut surgery is generally considered more technically challenging than primary surgery given the development of hiatal scarring and inflammation [10, 12]. While several single-institution studies have been conducted on the safety of MSA device removal, these studies are limited by small sample sizes [3, 11–14]. As such, it is valuable to consider a larger sample when analyzing the perioperative outcomes of MSA device removal. The objective of this study was to utilize the American College of Surgeons National Surgical Quality Improvement Program (ACS-NSQIP) registry to assess the short-term perioperative outcomes of patients undergoing MSA device explant with concurrent HHR and fundoplication compared to outcomes from primary HHR and fundoplication.

## Methods

### Data source and study design

A retrospective cohort study was performed using the ACS-NSQIP registry from 2017 to 2023. Data captured included outcomes up to 30 days postoperatively in addition to patient demographics, comorbidities, and operative details. The study was exempt from IRB approval as data are deidentified.

### Patient population

The study population included adults (18 years and older) undergoing foregut surgery during the study period. Foregut surgery was identified using CPT codes for specific procedure descriptions including 43,280 (laparoscopic esophagogastric fundoplasty), 43,281 and 43,282 (laparoscopic paraesophageal hernia repair with or without mesh, respectively), and 43,659 (unlisted laparoscopic gastric procedure). Explant of MSA devices was identified using CPT code 43,285. The aforementioned CPT codes were used to identify the cohort undergoing MSA device explant with concurrent hiatal hernia repair and fundoplication as well as the control cohort undergoing primary hiatal hernia repair and fundoplication.

### Variables and outcomes

Baseline characteristics included age, sex, body mass index (BMI), various comorbidities (including diabetes, steroid use, smoking status, etc.), and American Society of Anesthesiologists (ASA) classification.

The primary outcomes of interest were 30-day complications. Secondary outcomes included operative time, length of stay (LOS), 30-day readmission, 30-day reoperation, and 30-day mortality.

### Statistical analysis

Propensity score matching (PSM) was used to minimize confounding and selection bias. The cohort undergoing MSA device explant with HHR and fundoplication was matched 1:1 to the control cohort. Propensity scores were calculated using logistic regression with covariates including demographics, comorbidities, BMI, ASA classification, operative indication, and procedure type.

Continuous variables were compared using Mann–Whitney U test or *t* test. Similarly, categorical variables were compared using Chi-square test or Fisher’s exact test. Statistical significance was defined as *p* value < 0.05. Statistical analyses were performed in IBM SPSS Statistics (Version 30.0.0.0 (172)).

## Results

A total of 49,535 patients were identified as undergoing foregut surgery within the ACS-NSQIP database during the study period. Of these, 40 patients (0.01%) underwent concurrent MSA device explant. Following PSM, each cohort had 40 patients with comparable baseline characteristics as depicted in Table 1. There were no significant differences in the cohorts across age, gender, race, functional status, or comorbidities. Overall demographics between the MSA and primary hernia repair groups were similar with the majority being female (87.5%), a mean age of 58.3 and 58.8 years, and BMI of 29.1 and 28.9 kg/m2, respectively (Table 1).

**Table 1:** Comparison of baseline demographics of magnetic sphincter augmentation device explant with hiatal hernia repair and fundoplication (MSA) and primary hiatal hernia repair and fundoplication (primary) propensity score matched cohorts

		MSA *N* (%)	Primary *N* (%)	*p*-value
Gender				
Female		35 (87.5)	35 (87.5)	1.0
Male		5 (12.5)	5 (12.5)	1.0
Race				
Caucasian		35 (87.5)	35 (87.5)	1.0
African American		2 (5)	2 (5)	1.0
Asian		1 (2.5)	1 (2.5)	1.0
Unknown		2 (5)	2 (5)	1.0
Age (mean ± SD in years)		58.3 ± 14.7	58.8 ± 14.8	0.90
BMI (mean ± SD in kg/m^2^)		29.1 ± 6.33	28.9 ± 6.32	0.92
ASA				
II		17 (42.5)	17 (42.5)	1.0
III		22 (55)	22 (55)	1.0
IV		1 (2.5)	1 (2.5)	1.0
Diabetes		5 (12.5)	5 (12.5)	1.0
Current smoker		8 (20)	8 (20)	1.0
Independent functional status		40 (100)	40 (100)	1.0
COPD		1 (2.5)	1 (2.5)	1.0
Hypertension		17 (42.5)	17 (42.5)	1.0
Steroid use		1 (2.5)	1 (2.5)	1.0
Bleeding disorder		1 (2.5)	1 (2.5)	1.0

*SD* standard deviation, *ASA* American Society of Anesthesiologists classification, *COPD* Chronic obstructive pulmonary disease, *BMI* Body mass index

Analysis revealed that 30-day outcomes were not significantly different between cohorts as shown in Table 2. Neither cohort had any patients that developed surgical site infection, pneumonia, unplanned intubation, thromboembolic complications, acute renal failure, urinary tract infection, stroke, cardiac arrest, myocardial infarction, transfusion requirement, or sepsis. The majority (97.5%) of patients did not experience any complications, although one patient from each cohort required readmission for reoperation. There were no mortalities in either group (Table 2).

**Table 2:** Comparison of categorical outcomes of magnetic sphincter augmentation device explant with hiatal hernia repair and fundoplication (MSA) and primary hiatal hernia repair and fundoplication (primary) propensity score matched cohorts

	MSA *N* (%)	Primary *N* (%)	*p*-value
Surgical site infection	0	0	1.0
Pneumonia	0	0	1.0
Unplanned intubation	0	0	1.0
Pulmonary embolism	0	0	1.0
Acute renal failure	0	0	1.0
Urinary tract infection	0	0	1.0
Stroke	0	0	1.0
Cardiac arrest	0	0	1.0
Myocardial infarction	0	0	1.0
Need for transfusion	0	0	1.0
Deep vein thrombosis	0	0	1.0
Sepsis/septic shock	0	0	1.0
Unplanned reoperation	1 (2.5)	1 (2.5)	1.0
Readmission	1 (2.5)	1 (2.5)	1.0
Mortality	0	0	1.0

Median operative time for patients in the MSA device explant group was 135 min, while operative time for patients in the control cohort was 132 min. Median LOS was equivalent at 1 day for both groups (Table 3).

**Table 3:** Comparison of continuous outcomes of magnetic sphincter augmentation device explant with hiatal hernia repair and fundoplication (MSA) and primary hiatal hernia repair and fundoplication (primary) propensity score matched cohorts

	MSA Median [IQR]	Primary Median [IQR]	*p*-value
LOS	1 [1]	1 [1]	1.0
Operative time	135.5 [92, 171]	132 [92, 165]	0.86

*IQR* Interquartile range, *LOS* Length of stay

## Discussion

Our findings suggest that concurrent MSA device explant with HHR and fundoplication has a short-term safety profile similar to that of primary HHR and fundoplication. There were no significant differences in 30-day complications, operative time, LOS, reoperation, readmission, or mortality.

As the number of patients undergoing MSA device implantation increases, an increasing number of patients will require device explant. The most common indications for explant are intolerable dysphagia (38–77.5%), persistent GERD symptoms (20.5–54%), and less commonly serious complications such as device erosion or migration (0–8%) [3, 9, 11, 15]. Revisional foregut surgery is often challenging due to scarring and altered anatomy, often conferring increased operative time and complication rates [10, 12]. Our findings suggest that MSA device explantation and conversion to traditional fundoplication have few complications, similar to that of primary hiatal hernia repair and fundoplication. A fibrous capsule often forms around the MSA device, requiring incision of the capsule, division of the linking chain, and extracorporealization of the device. As a result, there is greater risk of esophageal and/or gastric injury during the dissection. The conversion to a fundoplication may, in addition to the management of gastroesophageal reflux control, provide a buttress to the underlying tissue, similar to that utilized during esophageal myotomy and fundoplication. Interestingly, the addition of MSA device explant did not significantly increase operative time over primary HHR and fundoplication. While the removal of the MSA device is more complex, the circumferential dissection of the gastroesophageal junction may facilitate hiatal hernia repair and clear the esophagus anteriorly and posteriorly for placement of a wrap. Alternatively, surgeons who are opting to remove a MSA device with concurrent hiatal hernia repair may also be more experienced than surgeons who opt for a staged approach.

Prior studies on MSA device explant have mainly been limited to single-institution studies. While our study did reveal a low rate of reoperation, Asti et al. found no major postoperative complications requiring reoperation among 11 patients requiring explant, all of whom reported postoperative satisfaction and normal Gastroesophageal Reflux Disease-Health-Related Quality of Life (GERD-HRQL) scores at last known follow-up [12]. Their study was based off of prospective data spanning 2007 to 2015 that included 164 patients who underwent MSA implantation; as such, they were additionally able to capture that the primary reason for device removal was symptom recurrence, pre-implant supine esophageal acid exposure was independently associated with explant, and explant primarily occurred at 1–2 years following implantation [12]. Another single-institution study by Tatum et al. found a mean operative time of 107 min in 24 device removals out of 435 implants, although with a heterogeneous sample given only 21% of these patients underwent concurrent fundoplication; the remainder of revisions underwent MSA replacement, gastrectomy, or MSA removal alone [11]. Their findings similarly reflected a short mean LOS of 1 day and lack of major complications [11]. Most similarly to our findings, Bona et al. found a median operative time of 145 min without significant complication, morbidity, or mortality among a sample of five device explants out of 32 implants from single-center prospectively collected data; however, LOS was higher with a median of 4 days and range of 3–6 days [13]. Similar to findings reported by Asti et al., postoperative GERD-HRQL and quality of life were improved [12, 13]. Although with small sample size, in their experience, they note that the most difficult dissection occurs on the posterior aspect of the esophagus due to the presence of adhesive disease [13]. It is somewhat surprising, then, that our findings suggest that operative time is comparable between our cohorts and may lend favor to the possibility of more experienced surgeons opting for a single-staged removal and revision approach.

Our study provides the first robust comparison on a national scale using PSM, the results of which are comparable to prior studies. Taken altogether, our findings suggest MSA can safely be considered without significant impact on future revisional HHR and fundoplication, should it be necessary. There were several limitations to consider in the context of these findings. Only short-term outcomes could be evaluated given that the ACS-NSQIP registry only captures 30-day outcomes. Conclusions regarding long-term outcomes cannot be drawn from the current study. Furthermore, the ACS-NSQIP registry does not provide granularity on the operative details nor quality-of-life outcomes. It would be beneficial to further assess the size of the associated hernia and the type of fundoplication that was utilized in each case to yield more informative results. Additionally, despite having a large national sample, the cohort size is still quite small and limits the statistical power of these results. Finally, given that individuals were identified based on CPT codes, variations in coding may have contributed to a smaller than expected cohort. It should also be noted that our study is not intended to compare the safety of MSA explant with concurrent HHR to revisional foregut surgery, but to assess additional risk conferred when using MSA devices and subsequently requiring removal and revision rather than proceeding directly to HHR.

## Conclusion

Magnetic sphincter augmentation device explant with concurrent revisional foregut surgery does not confer additional short-term perioperative risk when compared to patients undergoing primary HHR and fundoplication. Our findings suggest that the addition of MSA device explant does not impact short-term perioperative outcomes. Future studies are needed to evaluate quality-of-life metrics and long-term outcomes.
